# Identification of Sources of Resistance to Aphanomyces Root Rot in *Pisum*

**DOI:** 10.3390/plants13172454

**Published:** 2024-09-02

**Authors:** Sara Rodriguez-Mena, Diego Rubiales, Mario González

**Affiliations:** 1 Institute for Sustainable Agriculture, CSIC, 14004 Cordoba, Spain; mgonzalezromero@ias.csic.es; 2 Campus de Rabanales, University of Cordoba, 14014 Cordoba, Spain

**Keywords:** pea, root disease, *Aphanomyces euteiches*, aerial evaluation

## Abstract

Aphanomyces root rot (ARR), caused by *Aphanomyces euteiches*, is one of the most devastating diseases that affect the production of peas. Several control strategies such as crop rotation, biocontrol, and fungicides have been proposed, but none provides a complete solution. Therefore, the deployment of resistant cultivars is fundamental. ARR resistance breeding is hampered by the moderate levels of resistance identified so far. The available screening protocols require post-inoculation root assessment, which is destructive, time-consuming, and tedious. In an attempt to address these limitations, we developed a non-destructive screening protocol based on foliar symptoms and used it to identify new sources of resistance in a *Pisum* spp. germplasm collection. Accessions were root inoculated separately with two *A. euteiches* isolates, and leaf symptoms were assessed at 5, 10, 14, 17, and 20 days after inoculation (DAI). Although the majority of accessions exhibited high levels of susceptibility, thirty of them exhibited moderate resistance. These thirty accessions were selected for a second experiment, in which they were inoculated with both *A. euteiches* isolates at two inoculum doses. The objective of this second trial was to confirm the resistance of these accessions by evaluating root and biomass loss, as well as foliar symptoms, and to compare root and foliar evaluations. As a result, a high correlation (R^2^ = 0.75) between foliar and root evaluations was observed, validating the foliar evaluation method. Notably, accessions from *P*.*s*. subsp. *humile* exhibited the lowest symptomatology across all evaluation methods, representing valuable genetic resources for breeding programs aimed at developing pea varieties resistant to ARR.

## 1. Introduction

Legumes stand out as crops that are well suited for adaptation due to their symbiotic nitrogen fixation ability, which can contribute to sustainable agriculture [1,2]. Despite their adaptability, they are subject to a range of biotic and abiotic stresses, leading to significant yield reductions [3]. Among these challenges, Aphanomyces root rot (ARR), caused by the oomycete *Aphanomyces euteiches*, is one of the major diseases of legumes, affecting a variety of crops worldwide, including lentils (*Lens culinaris*), alfalfa (*Medicago sativa*), vetch (*Vicia sativa*), common beans (*Phaseolus vulgaris*) and, mainly, peas (*Pisum sativum*) [1,2,3,4]. This pathogen is present in many legume-growing areas, causing huge yield losses, including North America [4], Europe [5], Oceania [6], and Asia [7]. 

The primary inoculum of *A. euteiches* is oospores in the soil, which can remain viable for up to a decade. However, epidemics are mainly driven by the swift movement of biflagellate zoospores released by germinating oospores. These zoospores are chemically attracted to pea roots and, upon contact, shed their flagella, form cysts, and germinate, directly invading the root tissues and eventually causing leaf yellowing, plant stunting, and death [8,9]. The pathogen, which can infect plants at any stage, spreads rapidly in conditions of high soil moisture and temperatures between 22 and 28 °C. Early symptoms include water-soaked lesions on roots that turn honey-brown [7]. As a consequence of root malfunction, yellowing and necrosis start in the lower leaves and progress to the upper ones, resulting in plant death in the most severe cases [10].

The capacity of *A. euteiches* to produce resistance structures, along with its genetic diversity, makes it hard to control. Fungicides with ethaboxam and dinitroaniline-based products have shown the potential to suppress ARR, but their widespread adoption remains limited [11]. Alternative control methods, such as soil amendments or biocontrol, are being studied. Adding Brassicaceae family residues or lime to the soil can reduce the severity of Aphanomyces root rot (ARR), but these measures cannot eliminate the issue [12,13]. Several beneficial bacteria, including *Pseudomonas* spp., *Bacillus mycoides*, *Streptomyces* spp., *Rhizobium* spp., *Pantoaea agglomerans*, *Lysobacter capsica*, and *Burkholderia cepacian*, have shown activity against *A. euteiches* by inhibiting mycelial growth and/or zoospores’ germination, making them candidates as biocontrol agents [14,15,16]. Despite all of the above, no efficient in-crop or preventative treatment is available. The most effective strategies for managing this disease still rely on crop rotation and avoiding infected soils [17], although these are only short-term solutions due to the long-term viability of oospores in soil [18].

Focusing on genetic resistance seems to be crucial to finding a sustainable and effective way to manage this disease. Multiple germplasm screening programs have been developed, resulting in the identification of accessions with partial resistance [19,20,21,22,23]. Some of these sources of resistance were integrated into breeding programs to develop recombinant inbred lines (RILs) [19,24,25,26] and near-isogenic lines (NILs) [27]. This facilitated the identification of 27 meta-QTLs associated with *A. euteiches* resistance, grouped into seven main QTL regions [22,28,29], with five having weaker effects and the other two considered to be major [30,31]. However, the quantitative nature of this resistance complicates resistance breeding. Additionally, the identified QTLs are linked to unengaging agronomic features such as long internodes, anthocyanin production, or late flowering, making it necessary to continue research in this area [25,31].

The conventional approach to finding new sources of resistance involves large-scale screenings that conduct root evaluations 20 days post-inoculation [19,32]. However, this method involves rinsing the roots at the end of the experiments, which is both laborious and destructive, and can only be performed at a single timepoint. Some authors suggested complementing the root evaluation with a foliar assessment [24,25], but they did not examine the correlation between these two methods or the variations that arise when different isolates or doses are utilized. To address these questions, a comprehensive evaluation was conducted using a collection of 322 *Pisum* spp. accessions, comprising cultivars, landraces, and wild types, tested under controlled conditions against two *Aphanomyces* isolates. The principal objective of this study was to identify sources of resistance by analyzing various genotypes, based on a non-destructive aerial evaluation. This approach aimed to select potentially susceptible genotypes and demonstrate their tolerance or resistance in substrates artificially inoculated with higher inoculum pressures.

## 2. Results

### 2.1. Visual Screening According to Aerial Symptoms

**Figure 1:**
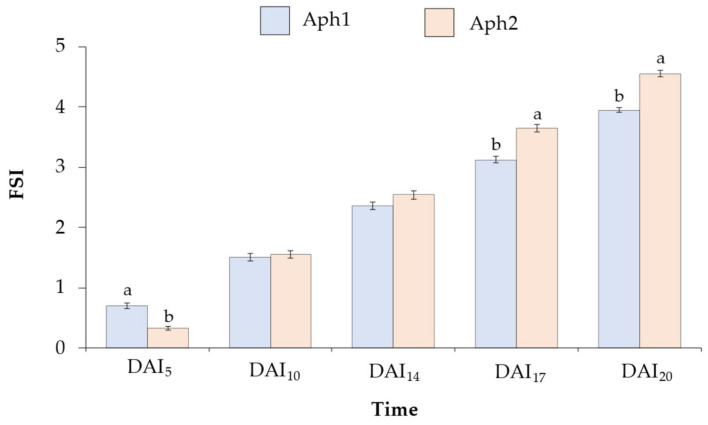
Average Foliar Symptoms Index (FSI) and Standard Error (SE) of a 322 *Pisum* collection at 5, 10, 14, 17, and 20 days after inoculation (DAI). The genotypes were inoculated with a 10^3^ zoospores/mL solution of two *A. euteiches* isolates: Aph1 (blue) and Aph2 (orange). FSI was assessed on a scale of 0 (healthy plant) to 5 (dead plant). For each time, bars with different letters (a or b) show significant differences between the two isolates according to the LSD test (α ≤ 0.05).

The aerial symptomatology caused by the two isolates of *A. euteiches* was assessed by evaluating the Foliar Symptoms Index (FSI) at 5, 10, 14, 17, and 20 days after inoculation (DAI) (Figure 1). To compare the response in terms of FSI caused by the two isolates of *A. euteiches*, an ANOVA was conducted. The results did not show significant differences between isolates at DAI_10_ and DAI_14_. Significant differences were observed between the two isolates at DAI_5_ (F = 44.4; *p* < 0.0001). However, the most significant differences between the isolates were observed at DAI_17_ (F = 35.1; *p* < 0.0001) and DAI_20_ (F = 71.4; *p* < 0.0001). The plants inoculated with Aph1 exhibited significantly lower levels of FSI_17_ (average 3.1 ± 0.1) and FSI_20_ (average 4.0 ± 0.0) compared to those inoculated with Aph2 (FSI_17_ = 3.6 ± 0.1; FSI_20_ = 4.5 ± 0.1). 

**Figure 2:**
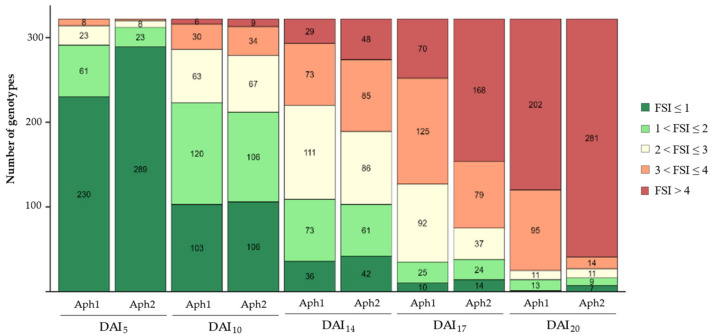
Distribution of genotypes according to Foliar Symptoms Index (FSI) at 5, 10, 14, 17, and 20 days after inoculation (DAI) of a 322 *Pisum* collection with a 10^3^ zoospores/mL solution of two *A. euteiches* isolates: Aph1 and Aph2. FSI was assessed on a scale of 0 (healthy plant) to 5 (dead plant). Colors represent FSI values for each genotype: dark green for FSI ≤ 1, light green for 1 < FSI ≤ 2, yellow for 2 < FSI ≤ 3, orange for 3 < FSI ≤ 4, and red for FSI > 4.

As shown in Figure 2, at DAI_5_, the majority of accessions (314 for Aph1 and 320 for Aph2) exhibited an FSI ≤ 3, indicating that, at this stage, the symptoms were not yet severe enough to observe clear differences between genotypes. By DAI_10_, there was a noticeable increase in symptom severity caused by both isolates, with the number of potentially resistant genotypes (FSI ≤ 3) decreasing to 286 for Aph1 and 279 for Aph2. From DAI_14_ onwards, the majority of genotypes were classified as susceptible (3 < FSI ≤ 4) or highly susceptible (FSI > 4). The reduction in low-severity genotypes (FSI ≤ 3) became more pronounced, reducing to 220 for Aph1 and 189 for Aph2 at DAI_14_, underscoring the increasing impact of both isolates, especially Aph2. Additionally, the number of genotypes considered to be potentially resistant decreased to 127 for Aph1 and 75 for Aph2 by DAI_17_. This trend was even more pronounced at the end of the experiment (DAI_20_), when 202 and 281 accessions were categorized as highly susceptible (FSI_20_ > 4) for Aph1 and Aph2, respectively. Only a few accessions remained in the lower symptom severity categories (25 for Aph1 and 27 for Aph2). These data underscore the potential for identifying resistant genotypes within the population. Consequently, DAI_20_ was considered optimal for discerning clear differences between accessions, and FSI_20_ was used as a parameter to differentiate between susceptible and potentially resistant genotypes.

**Figure 3:**
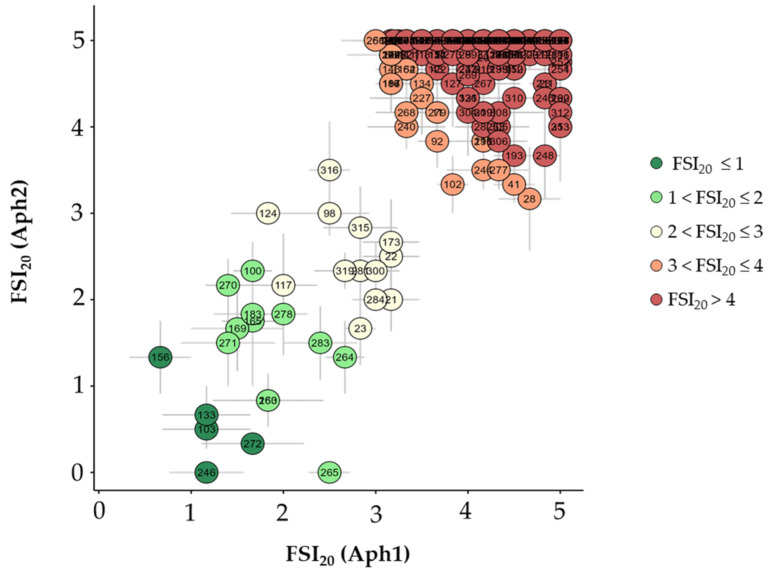
Average Foliar Symptoms Index 20 days after inoculation (FSI_20_) and Standard Error (SE) for the *Pisum* collection with 10^3^ zoospore/mL solutions of two *A. euteiches* isolates. The x-axis represents Aph1, and the y-axis represents the Aph2 isolate. FSI was assessed on a scale of 0 (healthy plant) to 5 (dead plant). Colors represent the mean FSI values for both isolates: dark green for FSI ≤ 1, light green for 1 < FSI ≤ 2, yellow for 2 < FSI ≤ 3, orange for 3 < FSI ≤ 4, and red for FSI > 4.

The susceptibility in terms of FSI to both isolates at DAI_20_ is shown in Figure 3. The majority of genotypes were highly susceptible to both isolates. Nonetheless, a total of 30 accessions showed a mean of FSI_20_ for both isolates equal to or below 3, which could indicate potential resistance to Aphanomyces root rot (Table S1). At this time, accessions 103, 133, 150, 156, 165, 169, 183, 246, 263, 271, and 272 displayed low aerial symptoms (FSI < 2) for both the Aph1 and Aph2 isolates. Accession 265 exhibited nearly zero symptoms for Aph2 and up to 2.5 FSI for Aph1, suggesting some isolate-specific resistant response. Additionally, other accessions (21, 22, 23, 98, 100, 117, 124, 173, 264, 270, 278, 281, 283, 284, 300, 315, 316, and 319) showed moderate symptom levels (2 < FSI_20_ ≤ 3) for both isolates.

**Figure 4:**
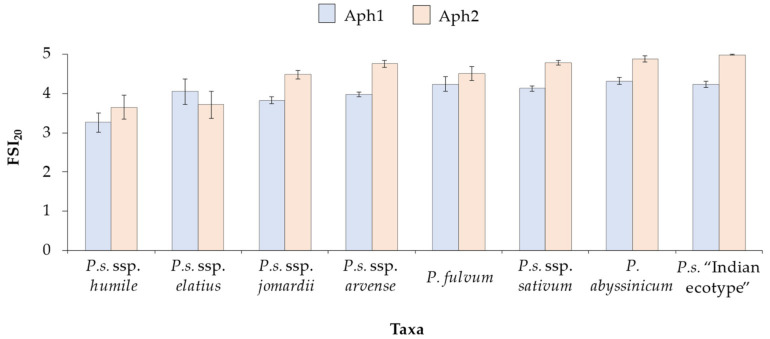
Average Foliar Symptoms Index 20 days after inoculation (FSI_20_) and Standard Error (SE) of 322 genotypes from the following taxa: *P.s*. subsp. *humile* (*n* = 28), *P.s*. subsp. *elatius* (*n* = 18), *P.s*. ssp. *jomardii* (*n* = 83), *P.s*. subsp. *arvense* (*n* = 81), *P. fulvum* (*n* = 13), *P.s*. subsp. *sativum* (*n* = 69), *P. abyssinicum* (*n* = 7), and *P. sativum* “Indian ecotype” (*n* = 23). The genotypes were inoculated with 10^3^ zoospores/mL of two *A. euteiches* isolates: Aph1 (blue) and Aph2 (orange), and FSI_20_ was assessed using a scale of 0 (healthy plant) to 5 (dead plant).

As shown in Figure 4, the results varied among taxa and accessions. *Pisum sativum* subsp. *humile* (*n* = 28) exhibited the majority of the most resistant accessions, with an average severity of 3.3 ± 0.2 for Aph1 and 3.7 ± 0.3 for Aph2. Ten accessions of this taxon were identified as resistant, including the most resistant ones: 272 (1.7 to Aph1 and 0.3 to Aph2), 133 (1.2 to Aph1 and 0.7 to Aph2), 271 (1.4 to Aph1 and 1.5 to Aph2), and 263 (1.8 to Aph1 and 0.8 to Aph2). Additionally, accessions 23, 100, 124, 165, 169, and 270 were considered to be moderately resistant. The group with the second-most potentially resistant accessions was *P.s*. subsp. *jomardii* (*n* = 83), showing an average severity of 3.8 ± 0.1 for Aph1 and 4.5 ± 0.1 for Aph2. Out of 83 accessions, 9 were identified as resistant. Notably, accession 156 was resistant to both Aph1 (0.7) and Aph2 (1.3), while accessions 150 (1.8 to Aph1 and 0.8 to Aph2) and 183 (1.7 to Aph1 and 1.8 to Aph2) were also resistant. Furthermore, accessions 117, 173, 281, 283, 315, and 319 were moderately resistant to both isolates. For *P.s*. subsp. *elatius* (*n* = 18) taxa, the average severity was 4.0 ± 0.3 for Aph1 and 3.7 ± 0.3 for Aph2. Four accessions were identified as resistant, with accessions 103 and 246 being the most resistant, while accessions 278 and 316 were moderately resistant. In *P.s*. subsp. *arvense* (*n* = 81), the average severity was 4.0 ± 0.1 for Aph1 and 4.8 ± 0.1 for Aph2, with four resistant accessions identified. Accessions 264 and 265 were moderately resistant to Aph1 (2.7 and 2.5, respectively) and highly resistant to Aph2 (1.3 and 0.0, respectively), with accessions 284 and 300 were moderately resistant to both isolates. For *P.s*. subsp. *sativum* (*n* = 69), the average severity was 4.1 ± 0.1 for Aph1 and 4.8 ± 0.1 for Aph2, with two accessions (21 and 22) showing FSI_20_ to Aph2 below 3. In *P. fulvum* (*n* = 13), the average severity was 4.2 ± 0.2 for Aph1 and 4.5 ± 0.2 for Aph2, with only one moderately resistant accession (98) showing scores of 2.5 for Aph1 and 3.0 for Aph2. Finally, no resistant accessions were identified in *P. abyssinicum* (*n* = 7) or in *P. sativum* “Indian ecotype” (*n* = 23).

The 30 potentially resistant accessions were selected for subsequent experimentation as potential sources of resistance to *A. euteiches*. In addition, due to the generally high level of aerial symptoms, nine additional susceptible accessions (68, 84, 163, 250, 279, 303, 306, 310, and 321) were added to the next experiment, and Messire plants were included as susceptible controls. 

### 2.2. Root and Foliar Symptoms of Selected Accessions

To verify the resistance of selected accessions as potential sources of resistance, six plants per accession were inoculated with two different zoospore suspensions (10^3^ and 10^4^ zoospores/mL) of both *A. euteiches* isolates. The foliar symptoms (FSI_20_) and Root Rot Index (RRI_20_) of each plant were assessed after 20 days. The general mean FSI_20_ caused by Aph1 at 10^3^ zoospores/mL was 2.3 ± 0.1, increasing to 2.9 ± 0.1 at the higher concentration of 10^4^ zoospores/mL. For Aph2, the mean FSI_20_ was 2.6 ± 0.1 at 10^3^ zoospores/mL and 3.5 ± 0.1 at 10^4^ zoospores/mL. Non-inoculated controls showed a low level of FSI_20_ (0.1 ± 0.0 on average). A similar and more pronounced pattern was observed in the RRI_20_, which, although reflecting the results from the FSI_20_, was more explanatory. The general average RRI_20_ caused by Aph1 at 10^3^ zoospores/mL was 5.5 ± 0.1, increasing to 6.3 ± 0.1 at the higher concentration of 10^4^ zoospores/mL. In the case of Aph2, the average RRI_20_ was 6.1 ± 0.1 at 10^3^ zoospores/mL, which further increased to 7.4 ± 0.1 at 10^4^ zoospores/mL. Control plants, which were not inoculated, showed low levels of RRI_20_ (0.2 ± 0.0 on average).

The relationship between root and foliar symptoms (RRI_20_ and FSI_20_) is represented in Figure S1, where the x-axis represents RRI_20_ and the y-axis denotes FSI_20_. The analysis revealed a consistent linear correlation, where an increase in RRI_20_ was directly proportional to an increase in FSI_20_, as indicated by the linear coefficient of determination (R^2^ = 0.75), according to the regression line y = 0.67·x−1.43. These values indicate that a substantial proportion of the variance in the RRI can be predicted from the FSI, signifying a robust predictive power for this linear model within the context of the genotypic response to *A. euteiches* infections.

**Figure 5:**
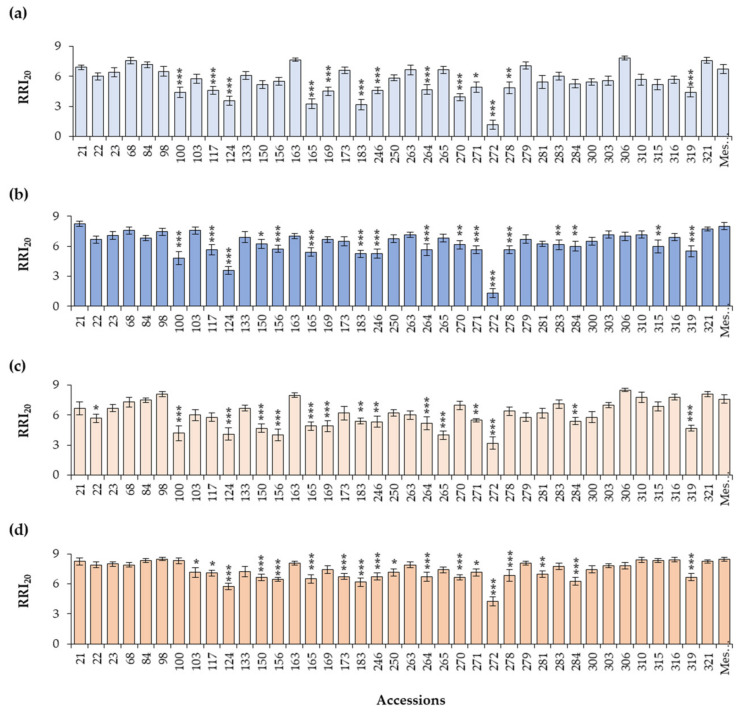
Average Root Rot Index and Foliar Index 20 days after the inoculation (RRI_20_) and Standard Error (SE) for the 40 selected accessions inoculated with both *A. euteiches* isolates: (**a**,**b**) inoculation with Aph1 at doses of 10^3^ zoospores/mL and 10^4^ zoospores/mL, respectively, and (**c**,**d**) Aph2 inoculations at doses of 10^3^ zoospores/mL and 10^4^ zoospores/mL, respectively. RRI_20_ was assessed on a scale of 0 (not root symptoms) to 9 (dead roots). Asterisks indicate significant differences (* *p* ≤ 0.1, ** *p* ≤ 0.05, and *** *p* ≤ 0.01) for each genotype with respect to the susceptible control Messire (Mes.).

The results of RRI_20_ are shown in Figure 5, and the specific results of foliar and root indices for each accession are presented in Table S2. These accessions were categorized as very resistant (VR), resistant (R), moderately resistant (MR), moderately susceptible (MS), susceptible (S), and very susceptible (VS), based on their root symptomatology indices (Table S2). Accession 272 was classified as resistant and exhibited the lowest values for both foliar and root symptoms for all zoospore concentrations, with RRI_20_ less than 2 for Aph1 and less than 4 for Aph2, even at the highest tested inoculum concentrations, indicating a notable level of resistance. In addition, two accessions were considered to be moderately resistant. Accession 124 showed low foliar (ranging from 0.6 to 2.2) and root symptoms values (ranging from 3.5 to 5.8), while accession 183 had consistently low FSI_20_ values (ranging from 1.1 to 2.3) and RRI_20_ values significantly lower than those of Messire, ranging from 3.2 for Aph1 at 10^3^ zoospores/mL to 6.2 for Aph2 at the highest dose. 

Thirteen accessions were considered to be moderately susceptible, exhibiting different levels of both foliar symptoms and root rot. Accessions 100, 117, and 165 showed low FSI_20_ and RRI_20_ values for all inoculum levels except for the highest dose of the Aph2 isolate, to which they were highly susceptible. Accessions 169, 270, and 319 were rated as resistant at the lower inoculum levels but susceptible at the highest doses of both isolates. Other accessions, such as 270 and 271, although displaying low FSI_20_ values, exhibited very high root symptoms. In addition, accessions 150, 156, 246, 264, and 284 showed moderate-to-high levels of symptoms for both isolates and doses. Some accessions, initially categorized as potentially resistant due to presenting an FSI_20_ value of 3 or lower, exhibited high or very high levels of root symptoms and were highly sensitive to increases in inoculum dose. This sensitivity led to their classification as susceptible (22, 103, 133, 173, 263, 265, 281, 283, 300, and 315) or very susceptible (21, 23, 98, and 316). Additionally, the accessions included as susceptible controls (250, 279, 303, 68, 84, 163, 306, 310, and 321) based on their FSI_20_ also demonstrated high susceptibility at the root level. In Figure S2, the phenotypic responses in the root and foliar symptoms of plants of accessions 272 (resistant), 124 (moderately resistant), and 306 (very susceptible) are shown, compared with the very susceptible control (Messire). 

### 2.3. Effect of the Inoculation in Wet Biomass 

**Figure 6:**
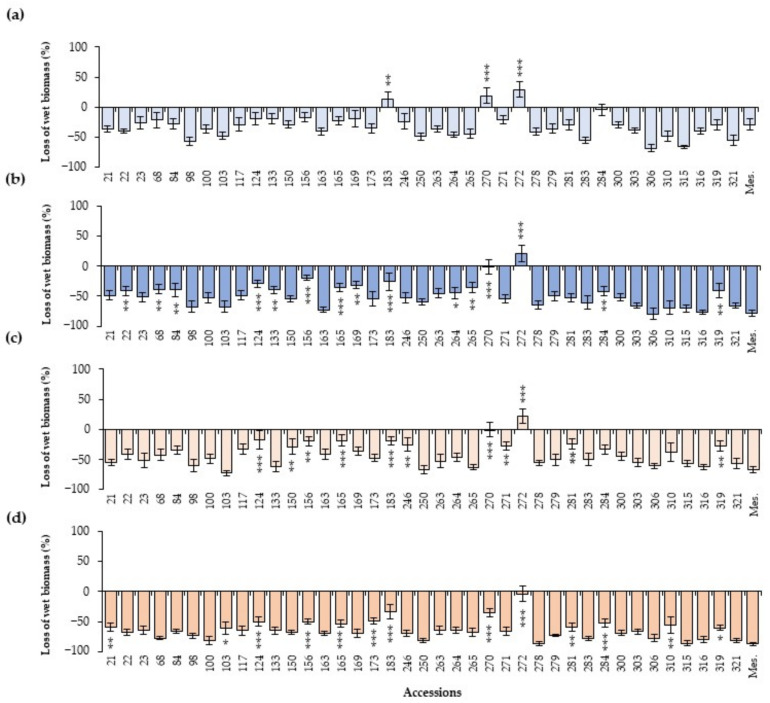
Average percentage of wet biomass loss with respect to the non-inoculated control of each accession 20 days after the inoculation, and Standard Error (SE), for the 40 selected accessions inoculated with both *A. euteiches* isolates: (**a**,**b**) inoculation with Aph1 at doses of 10^3^ zoospores/mL and 10^4^ zoospores/mL, respectively, and (**c**,**d**) Aph2 inoculations at doses of 10^3^ zoospores/mL and 10^4^ zoospores/mL, respectively. Asterisks indicate significant differences (* *p* ≤ 0.1, ** *p* ≤ 0.05, and *** *p* ≤ 0.01) for each genotype with respect to the susceptible control Messire (Mes.).

As summarized in Figure 6, wet biomass generally experienced a reduction when plants were inoculated with both isolates of *A. euteiches*. The general average wet biomass of plants inoculated with the 10^3^ zoospores/mL dose of Aph1 was a reduction of 30.8 ± 1.5% in biomass relative to the non-inoculated control, decreasing to a 41.6 ± 1.6% reduction in biomass at the higher concentration of 10^4^ zoospores/mL. This effect was most pronounced for the most virulent isolate Aph2, with a mean reduction of 48.7 ± 1.6% at 10^3^ zoospores/mL and 64.8 ± 1.1% at 10^4^ zoospores/mL. The majority of accessions exhibited a decrease in biomass weight when inoculated with *A. euteiches*. However, accession 272 did not show significant differences in wet weight when inoculated compared to the non-inoculated controls, while accessions 183 and 270 showed a reduction in wet biomass weight only in plants inoculated with Aph2 at the highest dose. Other accessions (124, 156, 165, 246, and 271) only showed a significant decrease in wet biomass when inoculated with Aph2. Accessions 133, 169, and 284 showed a similar biomass weight to the control in plants inoculated with Aph1 at 10^3^ zoospores/mL. The remaining accessions exhibited a very significant reduction in biomass weight for both isolates and both doses. 

## 3. Discussion

ARR is one of the major diseases that affect pea cultivation [4]. The long and high resistance of oospores in soils and the genetic diversity of this pathogen complicate its management [8,33]. At present, crop rotation is the most commonly employed method for controlling this disease, but it is not a viable long-term solution [11]. The development of resistant varieties is crucial to control ARR [22], and several breeding programs have been developed with this objective [19,24,26]. The traditional breeding programs are based on the assessment of resistance levels by evaluating root symptoms at the conclusion of the experiments at 2 weeks [34,35], 3–4 weeks [8,10], or 5 weeks [11,36]. These methods involve removing the plant from the soil, washing the roots, and scoring the extent of root rot. However, these methods are limited by reliance on a single evaluation point and further disadvantaged by the slow and labor-intensive process of individually washing each plant’s roots for assessment [25,31,37]. The present study allowed us to develop a rapid and non-destructive foliar method, as well as to identify new resistant sources from a *Pisum* collection comprising cultivars, landraces, and wild accessions tested against *A. euteiches*.

The temporal progression of foliar symptoms provided a detailed assessment of disease development. Initially, the absence of clear differences in FSI values across genotypes at DAI_5_, DAI_10_, and DAI_14_ indicated that the initial infection stages are relatively uniform across genotypes. This uniformity can be attributed to the fact that, at these early stages, primary symptoms occur at the root level, while the aerial symptoms manifested are secondary. The primary infection in the roots precedes the visible secondary symptoms on the foliage, showing a lag between the onset of root symptoms and their manifestation in the leaves [10]. In this study, the consistent increase in FSI values over time suggested that the root infections characteristically resulted in foliar symptoms only after a certain period, particularly the increase from DAI_17_ onwards, once the pathogen had significantly compromised the functionality of the root system. It was observed that assessments at DAI_20_ were particularly critical for accurately distinguishing between resistant and susceptible genotypes, demonstrating that, before DAI_20_, it is not entirely clear or reliable to obtain data on foliar symptoms that would allow for differentiation between susceptible and potentially resistant genotypes. At 20 days, the majority of the collection manifested high susceptibility to both isolates. Notably, the isolate Aph2 (RB84) demonstrated significant virulence, highlighting the aggressiveness of pathotype I isolates and reinforcing the need to include multiple isolates in resistance screening [38,39,40,41]. This finding aligns with previous studies indicating isolate-specific virulence differences in *A. euteiches*, underscoring the necessity of evaluating multiple isolates when screening for resistance [42]. 

Despite the overall high susceptibility, thirty of the evaluated accessions presented an average FSI_20_ lower than 3 between the two isolates, and these accessions were selected for a second trial. This experiment helped both to validate the foliar assessment method used initially and to facilitate a comparison of this aerial assessment with root symptom evaluations. A linear relationship with a high level of correlation (R^2^ = 0.75) between foliar symptoms and root symptoms was obtained, indicating that increases in root symptoms are directly proportional to increases in foliar symptoms. This substantial proportion of variance explained by the model validates the foliar evaluation conducted previously, demonstrating its effectiveness in assessing root rot severity and the phenotypic response to *A. euteiches* infections in the aerial part. This linear relationship reinforces the reliability of foliar symptom assessments as a predictive tool for root rot severity in breeding programs and resistance studies. In addition, the wet biomass of the selected plants was measured, observing a significant reduction in this parameter in the majority of the inoculated plants, particularly with the Aph2 isolate and at the highest doses of both isolates. Biomass weight has been also identified as an indicative parameter to assess peas’ resistance against ARR [43]. This measure reflects the overall response of the plant to the infection, involving resistant and tolerant components [44].

Although genetic resistance in peas could be the most economical and effective strategy for managing ARR, currently, no pea cultivars are fully resistant [5]. Different studies conducted in recent years focusing on the characterization and identification of alleles associated with partial resistance to ARR have shown the complicated genetics involved in resistance, making breeding more challenging [23,25,27,45]. However, it has been demonstrated that *Pisum* species and subspecies can cross and produce viable hybrids, which facilitates the exploitation of the wide genetic variation in peas during breeding [46,47,48]. 

In the last few decades, several pea accessions, such as Capella, MN144, MN313, MN314, 90-2131, 90-2079, 552, and PI 180693, have been described as partially resistant to certain strains of *A. euteiches* [19,24,26,43,49,50]. Among them, 552, and particularly PI 180693, have attracted significant interest due to their consistently high levels of partial resistance to this disease [43,51]. Specifically, the landrace PI 180693, initially identified as resistant by Lockwood [52], has been extensively studied due to its potential to tolerate *A. euteiches* infection [25,43], while also showing high levels of resistance to Fusarium root rot under controlled conditions [53,54,55]. However, it is considered to be resistant to ARR, although the resistance level depends on the tested isolate [43]. As part of our experiments, several genotypes showed a certain level of resistance to both isolates of *A. euteiches*, indicating their potential as sources of resistance against ARR. 

According to our results, the most resistant accessions, with root symptoms caused by the reference isolate Aph2 (RB84) less than 5, ordered from most resistant to moderately resistant, were 272 > 265 > 156 > 124 > 100 > 319 > 150 > 165 > 169. Among them, 100 (JI 85), 124 (JI 1107), 165 (CGN16582), 169 (CGN03328), and 272 (JI 1428) belong to *P.s*. subsp. *humile*, forming part of the Q6 cluster in the population structure analysis obtained by Rispail et al. [46]. These accessions, whose geographic origins comprise East Asia and South Asia, including China, Pakistan, Afghanistan, and Nepal, suggest a distinct genetic grouping within Q6. This classification correlates with their observed resistance levels, indicating potential sources of resistance that are geographically and genetically distinct. Notably, accession 272 emerged as the most resistant among all studied accessions. Initially, this accession was classified as *P*. *sativum* subsp. *tibetanicum*, similar to accession 270 (JI 804), which, despite showing moderately high root symptom levels, exhibited very low foliar symptom levels. These two accessions were the only ones that did not show a reduction in plant wet weight when inoculated with both isolates at the lowest doses. Accession 270 showed a reduction in wet biomass only when inoculated with the highest dose of the Aph2 isolate. The observation of very low foliar symptom levels could be agronomically interesting and might allow the plants to remain productive even when infected, which should be studied further in field trials. Therefore, accession 272 in particular, along with other interesting accessions such as 100, 124, 165, 169, and 270, should be considered very valuable for inclusion in breeding programs. These accessions could be used as sources of ARR resistance and genetic material for transferring resistance traits to agronomically desirable varieties [46,56,57,58].

Additionally, among the most resistant accessions were 150 (BGE023256), 156 (CGN03277), and 319. These accessions exhibited similar levels of root susceptibility to both isolates of *A. euteiches* and were classified as moderately susceptible. According to Rispail et al. [46], these accessions belong to the taxon *P.s*. subsp. *jomardii* and originated from different regions of Europe. It is noteworthy that accession 183 (PI 477372) was the only one that demonstrated resistance in the root evaluation for the isolate Aph1, despite being more susceptible to the reference isolate RB84. In addition, accession 265 (JI 199), classified as *P.s*. subsp. *arvense*, was considered moderately resistant to the isolate Aph2; however, it was more susceptible to the isolate Aph1. Both taxa, *P.s*. subsp. *jomardii* and *P.s*. subsp. *arvense*, emerged during pea domestication and form intermediate populations between wild and domesticated genotypes, having significant potential for pea breeding [46,59,60].

On the other hand, *P.s*. subsp. *elatius* has shown promise in breeding programs aimed at enhancing the nutritional content of peas and could still offer valuable genetic diversity for resistance to biotic stresses [61]. Among our selected genotypes of this taxon, genotype 246 (PI 273209) exhibited low foliar symptom levels despite having moderate root symptoms. Notably, this genotype has recently been identified as having a late-acting hypersensitive response against *Uromyces pisi* [62]. This suggests that *P.s*. subsp. *elatius* and, more specifically, this accession could represent an important source of partial resistance and hold potential for identifying new genomic regions linked to ARR resistance in peas. Wild pea relatives have already been successfully explored and utilized in pea breeding [56,57]. Their resistance traits have been effectively incorporated into pea cultivars [57,58], underscoring their significance as sources of valuable alleles in pea breeding. 

Several authors have reported high levels of resistance in *P*. *fulvum* accessions that showed high levels of resistance to rust caused by *U*. *pisi* [63], *Mycosphaerella pinodes* [64], *Erysiphe pisi* [65], *Orobanche crenata* [58,66,67], *Acyrthosiphon pisum* [68], *Bruchus pisorum* [69,70] and, recently, to race 2 of *Fusarium oxysporum* [71]. In contrast, in our work, only one accession of *P*. *fulvum* was initially identified as potentially moderately resistant (98; PI 595945), but in the second experiment, very high symptoms were observed at the root level, classifying it as very susceptible. Similarly, the two initially selected accessions 21 (PI 204305) and 22 (PI 204667), belonging to the taxon *P.s*. subsp. *sativum*, showed high root symptom levels, categorizing accession 22 as susceptible and 21 as very susceptible. Finally, no resistant accessions were found in *P*. *sativum* “Indian ecotype”. None of the tested accessions showed foliar symptom levels that allowed them to be classified as potentially resistant. Accession 68 (PI 347336), used as a control in the second experiment, exhibited very high root symptom levels, similar to those of the susceptible control Messire. A recent study has related the white-flowered accessions of peas with high susceptibility to root diseases caused by *Fusarium avenaceum* in different pea accessions, showing root rot and seedling death at early growth stages [72]. Our results suggested that genotypes with white flowers, such as those classified as *P*. *sativum* “Indian ecotype”, as well as many genotypes classified as *P*. *sativum* subsp. *sativum*, exhibited high susceptibility to *A*. *euteiches* [46]. 

The foliar evaluation method demonstrated a strong correlation with root symptoms, providing a rapid, non-destructive alternative for identifying resistant genotypes. Additionally, although the majority of inoculated accessions showed high susceptibility to *A. euteiches*, some resistant accessions maintained good root system health, which was also reflected in their foliar condition and wet weight, suggesting their ability to preserve the root system in response to infection. Among the evaluated accessions, the wild taxon *P.s*. subsp. *humile* emerged as a valuable source of resistance, positioning it as a potential candidate for exploring new sources of resistance against this pathogen. These observations underscore the importance of considering both foliar and root symptoms in breeding programs to develop robust, resistant pea varieties. Combining these strategies with the genetic potential of wild relatives can significantly enhance the development of durable, ARR-resistant pea cultivars. Future research should focus on field trials to validate these results and further explore the genetic mechanisms underlying resistance.

## 4. Materials and Methods

### 4.1. Plant Material

**Figure 7:**
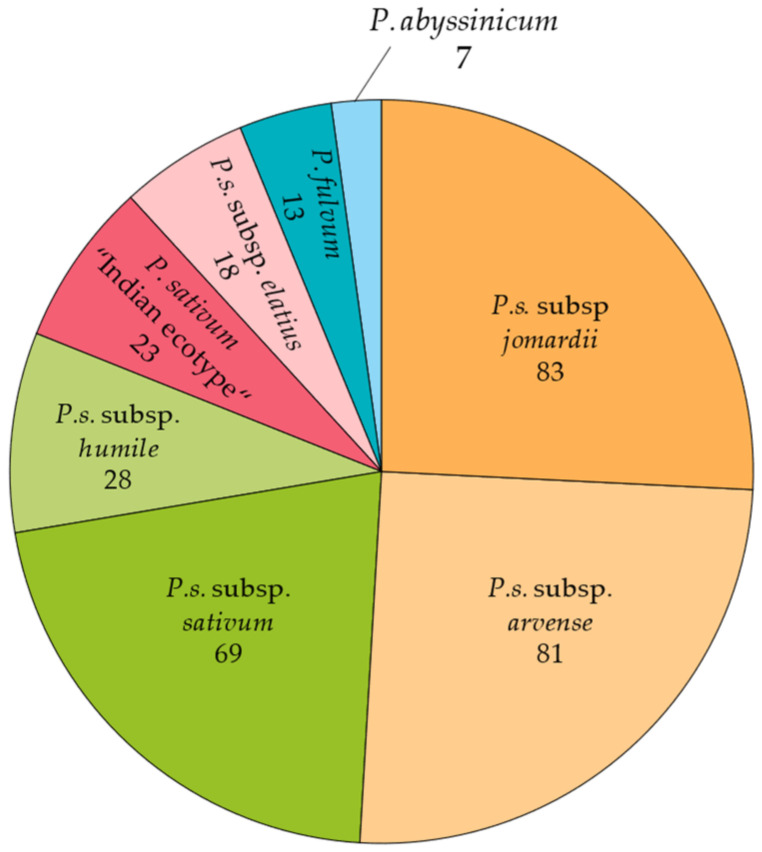
Taxonomic composition of the evaluated *Pisum* collection. The collection comprises 322 accessions from eight different *Pisum* taxa, including *P*.*s*. subsp. *jomardii* (86 accessions), *P*.*s*. subsp. *arvense* (81 accessions), *P*.*s*. subsp. *sativum* (69 accessions), *P*.*s*. subsp. *humile* (28 accessions), *P*. *sativum* “Indian ecotype” (23 accessions)*, P*.*s*. subsp. *elatius* (18 accessions), *P*. *fulvum* (13 accessions), and *P*. *abyssinicum* (7 accessions).

A core collection of 322 *Pisum* spp. accessions, including landraces, cultivars, and wild species with large genetic and morphologic diversity, was used across the experiments [46]. This collection was selected from a large *Pisum* spp. collection of more than 3000 accessions initially provided by the USDA (Department of Agriculture, USA), JIC (John Innes Center, UK), CRF-INIA (Centro Nacional de Recursos Fitogenéticos, Spain), CGN (CPRO-DLO, the Netherlands), IPK (Leibniz Institute of Plant Genetics and Crop Plant Research, Germany), and ICARDA (International Center for Agricultural Research in the Dry Areas, Syria). The collection is representative of the different *Pisum* species and subspecies, including accessions from *P.s*. subsp. *jomardii*, *P.s*. subsp. *arvense*, *P.s*. subsp. *sativum*, *P.s*. subsp. *humile*, *P. sativum* “Indian ecotype”, *P.s*. subsp. *elatius*, *P. fulvum*, and *P. abyssinicum* (Figure 7). While it does incorporate some commercial varieties and breeding lines, the majority of accessions consist of landraces, constituting 61% of the collection, with significant representation from wild species as well (16%) (Table S3). The cultivar Messire was used as a susceptible control.

For the experiments, seeds were sterilized with a 0.1% bleach solution, scarified, pregerminated, and sown in 200 mL plastic pots (6 × 6 × 10 cm) contained in plastic trays (8 × 6 pots per trays) and filled with autoclaved peat. Two seeds of each accession were sown in each pot. The experiment was set up in a randomized complete block design with three blocks, each including six trays. The pots were well watered every 3–4 days and incubated for 10 days in a growth chamber at the Institute of Sustainable Agriculture (24 ± 2 °C, 65 RH, 12:12 h D:N with 150 μmol m^−2^ s^−1^).

### 4.2. Aphanomyces euteiches Growth and Inoculation

Two isolates of *A. euteiches* were used for the experiment: Aph1 (UK), kindly provided by Dr. Lea Harold from PGRO (Processors and Growers Research Organisation; Peterborough, UK) [73], and the standard isolate Aph2 (RB84, France), pathotype I, provided by Dr. Marie-Laure Pilet-Nayet from INRAE (French National Institute for Agriculture, Food, and Environment; Rennes, France) [74]. Both isolates were separately grown on corn meal agar (CMA) medium for 7 days at 24 °C in the dark. Zoospores were produced by a modification of the method reported by Parke and Grau in 1992 [75]. Plugs (5 × 5 mm) from the advancing edge were transferred to Petri flasks containing 50 mL of peptone glucose (PG) broth (20 g/L oxide bacteriological peptone, 5 g/L glucose). Cultures were grown at 24 °C in the dark for 4 days, after which the PG broth was removed under sterile conditions, and mycelial mats were rinsed twice with sterilized water for 2 h. Then, 50 mL of mineral salt solution (0.26 g of CaCl_2_·2H_2_O, 0.07 g of KCl, and 0.49 g of MgSO_4·_7H_2_O dissolved in 1 L of sterile distilled water) was added to each flask and incubated at 24 °C in the dark for 20 h. During this time, zoospores were released into the water. Both inocula were separately filtered, and zoospore density was calculated using a Ross–Fushental hemocytometer. The concentrations of spores were adjusted to 10^3^ or 10^4^ zoospores/mL according to requirements [35]. Six plants of each genotype were inoculated by pipetting 5 mL of each isolate’s solution at the base of each plant. Additionally, six pots were inoculated with sterile water as controls.

### 4.3. Evaluation of Symptoms and Selection of Resistant Accessions

The Foliar Symptoms Index (FSI) was assessed using a scale from 0 to 5, as follows: 0 = healthy plant with no visible symptoms of wilting; 1 = slight (up to 20%) wilting of plant leaves; 2 = moderate, up to 40% of plant leaves and stem; 3 = disease progressed to above half of its height, with up to 60% of the plant, with most of the leaves and stem wilted and collar roots pulling out from the soil; 4 = extensive discoloration of plants, 80% of roots and stem wilted, with tissue collapse in soil; and 5 = entire plant collapsed, entire discoloration of the plant. Symptoms were evaluated 5, 10, 14, 17, and 20 days after the inoculation. 

The accessions that exhibited a Foliar Symptoms Index 20 days post-inoculation (FSI_20_) less than or equal to 3 were selected for a subsequent trial. In this experiment, six plants per genotype were independently inoculated with two inoculum doses (10^3^ and 10^4^ zoospores/mL) of both *A. euteiches* isolates (Aph1 and Aph2) to further assess the resistance of the selected accessions. Nine susceptible genotypes and Messire were used as susceptible controls. The Foliar Symptoms Index (FSI) was assessed using the same methodology as before, and root symptoms were measured using a 0–9 scale established by Xue (2000) [76] (Root Rot Index, RRI). For root evaluation, roots were carefully extracted from the pot. The peat was then discarded into a plastic bag, and the roots were meticulously washed in a water-filled plastic container. Based on the RRI, genotypes were classified as very resistant (0–3), resistant (3–4), moderately resistant (4–5), moderately susceptible (5–6), susceptible (6–7), and very susceptible (7–9). 

After the root evaluation, the aerial parts of the plants were cut and stored in airtight plastic bags at 4 °C. The following day, they were weighed to determine the wet biomass.

### 4.4. Data Analysis 

A general analysis of variance (ANOVA) was conducted to compare the FSI caused by both isolates at DAI_5_, DAI_10_, DAI_14_, DAI_17_, and DAI_20_, and means were compared by the LSD test at α = 0.05. Additionally, an ANOVA was conducted to compare the FSI_20_ and RRI_20_ data of the selected genotypes. The effect of the inoculum on each genotype was assessed by comparing mean values with the positive control (Messire) using a multiple comparisons test to detect differences less than the control, with significance levels set at α = 0.1, α = 0.05, and α = 0.01. In addition, the percentage of wet biomass loss was calculated as the percentage that the biomass of the treated genotype represented relative to the biomass of the untreated control, which was considered to be 100%. The effect of the inoculation on the percentage of wet biomass loss was also tested using a multiple comparisons test to detect differences higher than the positive control (Messire), with significance levels set at α = 0.1, α = 0.05, and α = 0.01. All statistical analyses were carried out using Statistix 9.0 software (Tallahassee, FL, USA). Graphs were created using R 4.2.3 software.

## Data Availability

Any required data that support the findings are available from the corresponding author upon request.

## Supplementary Materials

The following supporting information can be downloaded at: https://www.mdpi.com/article/10.3390/plants13172454/s1, Table S1: List of the accessions presenting a Foliar Symptoms Index 20 days after the inoculation (FSI_20_) of less than 3.5 with a 10^3^ zoospores/mL dose for at least one isolate. Includes ID, bank code, taxa, and the average FSI_20_ ± SE for the two *A. euteiches* isolates, Aph1 and Aph2. Table S2: Average Foliar Symptoms Index (FSI_20_) and Root Rot Index (RRI_20_) ± SE for the 40 selected accessions inoculated with both isolates (Aph1 and Aph2) at 10^3^ and 10^4^ zoospores/mL. Asterisks indicate values significantly different from the susceptible control Messire (* *p* ≤ 0.1, ** *p* ≤ 0.05, and *** *p* ≤ 0.01). Table S3: Accession list of the *Pisum* collection, including ID, bank code, species, population structure, origin, and material type. Figure S1: Relationship between the Foliar Symptoms Index (FSI_20_) (y-axis) and Root Rot Index (RRI_20_) (x-axis) of the 40 selected accessions 20 days after the inoculation. Blue dots represent the average values of accessions inoculated with the Aph1 isolate, with light blue for the 10^3^ zoospores/mL solution and dark blue for the 10^4^ zoospores/mL solution. Orange dots represent the average values of accessions inoculated with the Aph2 isolate, with light orange for the 10^3^ zoospores/mL solution and dark orange for the 10^4^ zoospores/mL solution. The adjusted regression line is y = 0.67·x −1.43 (R^2^ = 0.75). Figure S2: Phenotypic responses of various accessions (272, 124, 306, and the susceptible control Messire) to inoculation with two isolates of *A. euteiches* (Aph1 and Aph2). (a-t) Individual accessions subjected to different concentrations of inoculum. Uninoculated plants are shown in the first column, followed by Aph1 inoculated at doses of 10^3^ and 10^4^ zoospores/mL in the second and third columns, respectively, and Aph2 inoculation at the same doses in the fourth and fifth columns, respectively. This figure visually demonstrates the range of genotype reactions, from resistance (272 and 124) to susceptibility (306) compared with the susceptible control (Messire), under experimental conditions.
