# Supplementary material for: Identification of Sources of Resistance to Aphanomyces Root Rot in Pisum
Source: Plants (Basel). 2024 Sep 2;13(17):2454. doi: 10.3390/plants13172454 (PMC11397196; doi:10.3390/plants13172454)
Supplement: Supplementary file 1 [file plants-13-02454-s001.zip › plants-3132896-supplementary.pdf]

Table S1. List of the accessions presenting a Foliar Symptoms Index 20 days after the inoculation (FSI_{20}) of less than 3.5 with a 10^3 zoospores/mL dose for at least one isolate. Includes ID, bank code, taxa, and the average FSI_{20} ± SE for the two *A. euteiches* isolates, Aph1 and Aph2.

ID	Reference	Taxa	FSI_{20}	
			**Aph1**	**Aph2**
8	PI 143483	*P.s.* subsp. *sativum*	3.2±0.3	5.0±0.0
21	PI 204305	*P.s.* subsp. *sativum*	3.2±0.3	2.0±0.4
22	PI 204667	*P.s.* subsp. *sativum*	3.2±0.3	2.5±0.2
23	PI 220175	*P.s.* subsp. *humile*	2.8±0.2	1.7±0.4
24	PI 220673	*P.s.* subsp. *arvense*	3.2±0.3	5.0±0.0
25	PI 222069	*P.s.* subsp. *humile*	3.3±0.3	5.0±0.0
28	PI 254626	*P.s.* subsp. *jomardii*	4.7±0.3	3.2±0.6
41	PI 306592	*P.s.* subsp. *jomardii*	4.5±0.2	3.3±0.2
63	PI 347330	*P. sativum* "Indian ecotype"	3.2±0.3	4.8±0.2
70	PI 347342	*P.s.* subsp. *arvense*	3.5±0.2	5.0±0.0
81	PI 347373	*P.s.* subsp. *arvense*	3.2±0.2	5.0±0.0
82	PI 347374	*P.s.* subsp. *arvense*	3.3±0.2	4.8±0.2
83	PI 347375	*P.s.* subsp. *arvense*	3.2±0.3	5.0±0.0
88	PI 347401	*P. sativum* "Indian ecotype"	3.5±0.3	5.0±0.0
98	PI 595945	*P. fulvum*	2.5±0.4	3.0±0.3
100	JI 85	*P.s.* subsp. *humile*	1.7±0.2	2.3±0.3
102	JI 156	*P.s.* subsp. *jomardii*	3.8±0.2	3.3±0.3
103	JI 262	*P.s.* subsp. *elatius*	1.2±0.5	0.5±0.2
104	JI 263	*P.s.* subsp. *jomardii*	3.2±0.2	4.5±0.3
105	JI 228	*P.s.* subsp. *arvense*	3.2±0.2	5.0±0.0
107	JI 209	*P.s.* subsp. *jomardii*	3.5±0.3	5.0±0.0
110	JI 196	*P.s.* subsp. *humile*	3.5±0.2	5.0±0.0
111	JI 190	*P.s.* subsp. *jomardii*	3.5±0.2	5.0±0.0
115	JI 268	*P.s.* subsp. *jomardii*	3.5±0.2	5.0±0.0
116	JI 275	*P.s.* subsp. *arvense*	3.2±0.2	4.5±0.3
117	JI 280	*P.s.* subsp. *jomardii*	2.0±0.4	2.2±0.6
118	JI 288	*P.s.* subsp. *arvense*	3.5±0.4	4.8±0.2
123	JI 1089	*P.s.* subsp. *jomardii*	3.2±0.2	5.0±0.0
124	JI 1107	*P.s.* subsp. *humile*	1.8±0.4	3.0±0.0
132	JI 2387	*P.s.* subsp. *arvense*	3.3±0.2	5.0±0.0
133	JI 2545	*P.s.* subsp. *humile*	1.2±0.5	0.7±0.3
134	BGE001004	*P.s.* subsp. *jomardii*	3.5±0.2	4.5±0.5
140	BGE002168	*P.s.* subsp. *jomardii*	3.2±0.3	5.0±0.0
141	BGE003315	*P.s.* subsp. *arvense*	3.3±0.2	4.8±0.2
144	BGE004958	*P.s.* subsp. *jomardii*	3.3±0.3	5.0±0.0
146	BGE006126	*P.s.* subsp. *sativum*	3.2±0.2	4.7±0.3
147	BGE019594	*P.s.* subsp. *arvense*	3.5±0.2	5.0±0.0
149	BGE020326	*P.s.* subsp. *arvense*	3.2±0.5	4.8±0.2
150	BGE023256	*P.s.* subsp. *jomardii*	1.8±0.3	0.8±0.3
154	BGE026428	*P.s.* subsp. *sativum*	3.3±0.2	4.7±0.2
156	CGN16690	*P.s.* subsp. *jomardii*	0.7±0.3	1.3±0.4
159	CGN16640	*P.s.* subsp. *jomardii*	3.2±0.5	5.0±0.0
160	CGN16562	*P.s.* subsp. *humile*	3.2±0.2	5.0±0.0
162	CGN16581	*P.s.* subsp. *jomardii*	3.3±0.2	4.7±0.2
165	CGN16582	*P.s.* subsp. *humile*	1.7±0.3	1.7±0.7
169	CGN03328	*P.s.* subsp. *humile*	1.5±0.5	1.7±0.5
170	CGN03170	*P.s.* subsp. *arvense*	3.5±0.2	5.0±0.0
171	CGN03190	*P.s.* subsp. *jomardii*	3.3±0.2	5.0±0.0
172	CGN03245	*P.s.* subsp. *arvense*	3.2±0.3	4.8±0.2
173	CGN03165	*P.s.* subsp. *jomardii*	3.2±0.3	2.7±0.5
175	CGN03171	*P.s.* subsp. *jomardii*	3.5±0.2	5.0±0.0
182	PI 413686	*P.s.* subsp. *sativum*	3.2±0.2	5.0±0.0
183	PI 477371	*P.s.* subsp. *jomardii*	1.7±0.2	1.8±0.5
187	PI 324705	*P.s.* subsp. *jomardii*	3.2±0.2	4.5±0.3
192	PI 198074	*P.s.* subsp. *jomardii*	3.2±0.3	5.0±0.0
194	PI 357293	*P.s.* subsp. *sativum*	3.2±0.3	4.8±0.2
195	PI 249645	*P.s.* subsp. *arvense*	3.2±0.4	5.0±0.0
197	PI 357289	*P.s.* subsp. *sativum*	3.3±0.2	5.0±0.0

207	JI 2302	*P.s.* subsp. *sativum*	3.3±0.4	5.0±0.0
214	Kagpa	*P.s.* subsp. *sativum*	3.2±0.2	5.0±0.0
216	Pinochio	*P.s.* subsp. *sativum*	3.2±0.5	5.0±0.0
220	JI 1210	*P.s.* subsp. *sativum*	3.2±0.2	5.0±0.0
227	JI 82	*P.s.* subsp. *humile*	3.5±0.6	4.3±0.4
233	W6 17517	*P.s.* subsp. *sativum*	3.2±0.2	5.0±0.0
235	W6 17520	*P.s.* subsp. *sativum*	3.3±0.4	5.0±0.0
240	BGE023667	*P.s.* subsp. *jomardii*	3.3±0.4	4.0±0.3
244	PI 173055	*P.s.* subsp. *elatius*	4.2±0.4	3.5±0.2
246	PI 273209	*P.s.* subsp. *elatius*	1.2±0.4	0.0±0.0
247	PI 344003	*P.s.* subsp. *arvense*	3.2±0.4	4.8±0.2
256	PI 505127	*P.s.* subsp. *jomardii*	3.0±0.3	5.0±0.0
261	PI 505111	*P.s.* subsp. *jomardii*	3.0±0.4	5.0±0.0
263	JI 45	*P.s.* subsp. *humile*	1.8±0.6	0.8±0.3
264	JI 198	*P.s.* subsp. *arvense*	2.7±0.2	1.3±0.4
265	JI 199	*P.s.* subsp. *arvense*	2.5±0.2	0.0±0.0
270	JI 804	*P.s.* subsp. *humile*	1.4±0.2	2.2±0.3
271	JI 1398	*P.s.* subsp. *humile*	1.4±0.5	1.5±0.5
272	JI 1428	*P.s.* subsp. *humile*	1.7±0.6	0.3±0.3
277	CGN10205	*P.s.* subsp. *elatius*	4.3±0.3	3.5±0.2
278	CGN10206	*P.s.* subsp. *elatius*	2.0±0.3	1.8±0.5
281	IFPI 3370	*P.s.* subsp. *jomardii*	2.8±0.4	2.3±0.5
283	IFPI 436	*P.s.* subsp. *jomardii*	2.4±0.4	1.5±0.4
284	IFPI 2348	*P.s.* subsp. *arvense*	3.0±0.3	2.0±0.0
300	IFPI 2370	*P.s.* subsp. *arvense*	3.0±0.3	2.3±0.3
303	IFPI 2441	*P.s.* subsp. *jomardii*	3.5±0.2	5.0±0.0
315	IFPI 3330	*P.s.* subsp. *jomardii*	2.8±0.4	2.8±0.5
316	IFPI 3334	*P.s.* subsp. *elatius*	2.5±0.2	3.5±0.6
319		*P.s.* subsp. *jomardii*	2.7±0.3	2.3±0.2
Rest of accesions			>3.5	>3.5

Table S2. Average Foliar Symptoms Index (FSI_{20}) and Root Rot Index (RRI_{20}) ± Standard Error (SE) for the 40 selected accessions inoculated with both isolates (Aph1 and Aph2) at 10^3 and 10^4 zoospores/mL. Asterisks indicate values significantly different from the susceptible control Messire (* $p \leq 0.1$, ** $p \leq 0.05$ and *** $p \leq 0.01$).

ID	Taxa	Class (-)	FSI20				RRI20			
			Aph1		Aph2		Aph1		Aph2	
			10^3	10^4	10^3	10^4	10^3	10^4	10^3	10^4
21	*P.s.* subsp. *sativum*	(VS)	2.7±0.3	3.4±0.2	3.2±0.1	4.2±0.2	6.9±0.2	8.2±0.2	6.7±0.7	8.2±0.4
22	*P.s.* subsp. *sativum*	(S)	3.1±0.3	3.2±0.3	2.2±0.2***	3.8±0.3	6.0±0.4	6.7±0.4	5.7±0.4*	7.9±0.3
23	*P.s.* subsp. *humile*	(VS)	3.0±0.4	3.2±0.4	2.5±0.4**	3.4±0.4	6.4±0.5	7.1±0.4	6.7±0.4	8.0±0.2
68	*P. s.* “Indian ecotype”	(VS)	3.9±0.2	4.0±0.2	4.2±0.2	4.2±0.2	7.6±0.3	7.6±0.4	7.3±0.5	7.9±0.2
84	*P.s.* subsp. *arvense*	(VS)	3.8±0.2	3.6±0.1	4.3±0.3	4.1±0.2	7.2±0.3	6.8±0.2	7.5±0.2	8.3±0.2
98	*P. fulvum*	(VS)	3.2±0.4	3.9±0.3	4.1±0.3	4.7±0.1	6.5±0.5	7.5±0.4	8.1±0.3	8.5±0.2
100	*P.s.* subsp. *humile*	(MS)	2.3±0.3	1.8±0.3***	2.0±0.4***	4.4±0.3	4.4±0.5***	4.8±0.7***	4.2±0.7***	8.3±0.3
103	*P.s.* subsp. *elatius*	(S)	2.0±0.3*	3.9±0.4	1.0±0.4***	4.0±0.3	5.8±0.5	7.6±0.3	6.0±0.5	7.2±0.5*
117	*P.s.* subsp. *jomardii*	(MS)	0.8±0.2***	1.4±0.4***	1.8±0.5***	2.2±0.5***	4.6±0.4***	5.7±0.5***	5.8±0.4	7.1±0.3*
124	*P.s.* subsp. *humile*	(MR)	0.6±0.3***	1.1±0.4***	1.3±0.3***	2.2±0.1***	3.5±0.5***	3.6±0.4***	4.1±0.6***	5.8±0.3***
133	*P.s.* subsp. *humile*	(S)	2.1±0.1*	2.8±0.5**	2.3±0.3***	3.0±0.5***	6.1±0.4	6.9±0.6	6.7±0.3	7.2±0.5
150	*P.s.* subsp. *jomardii*	(MS)	2.5±0.3	3.2±0.3	2.2±0.2***	2.8±0.3***	5.2±0.4	6.2±0.5*	4.7±0.4***	6.7±0.3***
156	*P.s.* subsp. *jomardii*	(MS)	1.5±0.4***	1.8±0.3***	1.4±0.2***	2.3±0.3***	5.5±0.4	5.8±0.4***	4.0±0.6***	6.5±0.2***
163	*P.s.* subsp. *arvense*	(VS)	3.7±0.3	3.5±0.2	4.2±0.1	4.4±0.2	7.7±0.1	7.0±0.3	8.0±0.2	8.1±0.2
165	*P.s.* subsp. *humile*	(MS)	0.4±0.1***	1.4±0.3***	0.7±0.2***	1.7±0.4***	3.2±0.5***	5.4±0.4***	4.9±0.4***	6.5±0.4***
169	*P.s.* subsp. *humile*	(MS)	1.7±0.5***	3.3±0.2	1.9±0.3***	3.9±0.3	4.5±0.4***	6.7±0.3	4.9±0.5***	7.4±0.4
173	*P.s.* subsp. *jomardii*	(S)	2.5±0.4	3.1±0.3	2.7±0.5*	3.1±0.3***	6.6±0.3	6.5±0.5	6.2±0.7	6.8±0.3***
183	*P.s.* subsp. *jomardii*	(MR)	1.1±0.3***	2.3±0.3***	2.0±0.3***	2.2±0.4***	3.2±0.5***	5.2±0.4***	5.4±0.3**	6.2±0.4***
246	*P.s.* subsp. *elatius*	(MS)	1.3±0.3***	2.4±0.2***	2.2±0.2***	3.3±0.3*	4.6±0.3***	5.3±0.4***	5.3±0.6**	6.8±0.4***
250	*P.s.* subsp. *elatius*	(S)	3.3±0.3	3.6±0.3	3.8±0.2	3.9±0.3	5.8±0.3	6.8±0.4	6.2±0.3	7.2±0.4*
263	*P.s.* subsp. *humile*	(S)	2.3±0.4	3.2±0.5	2.6±0.3*	3.5±0.3	6.6±0.5	7.2±0.3	6.0±0.4	7.9±0.3
264	*P.s.* subsp. *arvense*	(MS)	1.5±0.4***	3.3±0.3	1.8±0.4***	2.9±0.4***	4.7±0.5***	5.7±0.6***	5.2±0.7***	6.7±0.4***
265	*P.s.* subsp. *arvense*	(S)	2.6±0.5	3.2±0.3	0.5±0.2***	4.1±0.3	6.6±0.3	6.8±0.4	4.0±0.4***	7.4±0.3
270	*P.s.* subsp. *humile*	(MS)	1.3±0.1***	1.6±0.4***	2.1±0.4***	2.1±0.3***	3.9±0.3***	6.2±0.4**	7.0±0.4	6.7±0.3***

271	*P.s.* subsp. *humile*	(MS)	1.5±0.3***	1.8±0.3***	1.7±0.3***	2.8±0.4***	4.9±0.5*	5.7±0.4***	5.5±0.2**	7.2±0.4*
272	*P.s.* subsp. *humile*	(VR)	0.5±0.2***	0.4±0.1***	0.5±0.3***	1.3±0.3***	1.2±0.5***	1.3±0.4***	3.2±0.6***	4.2±0.4***
278	*P.s.* subsp. *elatius*	(MS)	1.8±0.4**	2.8±0.2*	1.9±0.3***	4.0±0.3	4.8±0.6**	5.7±0.4***	6.4±0.4	6.8±0.6***
279	*P.s.* subsp. *jomardii*	(S)	3.8±0.2	3.7±0.2	2.7±0.5*	3.7±0.1	7.1±0.3	6.7±0.4	5.8±0.4	8.1±0.2
281	*P.s.* subsp. *jomardii*	(S)	2.1±0.4*	1.8±0.3***	1.9±0.2***	2.8±0.3***	5.5±0.6	6.2±0.3	6.2±0.5	7.0±0.3**
283	*P.s.* subsp. *jomardii*	(S)	2.8±0.3	3.7±0.2	2.9±0.2	3.8±0.3	6.0±0.4	6.2±0.5**	7.1±0.4	7.8±0.3
284	*P.s.* subsp. *arvense*	(MS)	2.2±0.3*	2.9±0.3	2.4±0.2***	3.5±0.2	5.2±0.4	6.0±0.5**	5.4±0.3**	6.2±0.4***
300	*P.s.* subsp. *arvense*	(S)	2.4±0.3	3.2±0.3	2.6±0.3*	3.9±0.4	5.4±0.3	6.5±0.4	5.8±0.6	7.5±0.3
303	*P.s.* subsp. *jomardii*	(S)	2.3±0.1	3.8±0.2	4.3±0.3	3.8±0.2	5.6±0.5	7.2±0.3	7.0±0.3	7.8±0.2
306	*P.s.* subsp. *jomardii*	(VS)	3.7±0.2	3.8±0.2	4.3±0.3	4.4±0.2	7.8±0.2	7.0±0.4	8.5±0.2	7.8±0.3
310	*P. fulvum*	(VS)	3.9±0.3	4.3±0.3	4.8±0.1	4.6±0.1	5.7±0.5	7.2±0.4	7.8±0.5	8.4±0.3
315	*P.s.* subsp. *jomardii*	(S)	2.5±0.3	2.8±0.2*	3.1±0.3	4.2±0.3	5.2±0.5	6.0±0.6**	6.9±0.5	8.4±0.2
316	*P.s.* subsp. *elatius*	(VS)	2.1±0.3*	3.2±0.3	4.2±0.3	4.3±0.2	5.7±0.4	6.9±0.4	7.8±0.3	8.4±0.2
319	*P.s.* subsp. *jomardii*	(MS)	1.7±0.3***	2.6±0.4**	1.8±0.3***	2.7±0.3***	4.4±0.5***	5.5±0.6***	4.7±0.3***	6.7±0.4***
321	*P.s.* subsp. *sativum*	(VS)	3.4±0.3	3.8±0.2	4.2±0.2	4.3±0.2	7.6±0.3	7.8±0.2	8.1±0.2	8.2±0.2
Messire	*P.s.* subsp. *sativum*	(VS)	3.5±0.3	4.1±0.3	4.0±0.3	4.6±0.2	6.8±0.5	8.0±0.4	7.6±0.4	8.5±0.2

(-) Class of susceptibility: Very Resistant (VR) (RRI$_{20}$ 0 to 3), Resistant (R) (RRI$_{20}$ 3 to 4), Moderately Resistant (MR) (RRI$_{20}$ 4 to 5), Moderately Susceptible (MS) (RRI$_{20}$ 5 to 6), Susceptible (S) (RRI$_{20}$ 6 to 7) and Very Susceptible (VS) (RRI$_{20}$ 8 to 9).

Table S3. Accession list of the *Pisum* collection including ID, bank code, species, population structure and origin.

ID	Reference	Taxa	Structure[1]	Origin
1	PI 109865	*P.s.* subsp. *arvense*	Adm.	Venezuela
2	PI 117910	*P.s.* subsp. *sativum*	Q3	Brazil
3	PI 140297	*P.s.* subsp. *sativum*	Adm.	Iran
4	PI 142442	*P.s.* subsp. *arvense*	Adm.	Peru
5	PI 142774	*P.s.* subsp. *jomardii*	Adm.	Mexico
6	PI 142776	*P.s.* subsp. *arvense*	Adm.	Guatemala
7	PI 142776	*P.s.* subsp. *arvense*	Adm.	Guatemala
8	PI 143483	*P.s.* subsp. *sativum*	Adm.	Iran
9	PI 143484	*P.s.* subsp. *jomardii*	Adm.	Iran
10	PI 143486	*P.s.* subsp. *arvense*	Adm.	Iran
11	PI 153351	*P.s.* subsp. *arvense*	Adm.	Ecuador
12	PI 162568	*P.s.* subsp. *sativum*	Q3	Argentina
13	PI 162692	*P.s.* subsp. *jomardii*	Adm.	Argentina
14	PI 162693	*P.s.* subsp. *arvense*	Adm.	Argentina
15	PI 162693	*P.s.* subsp. *arvense*	Adm.	Argentina
16	PI 162910	*P.s.* subsp. *sativum*	Q3	Paraguay
17	PI 164568	*P.s.* subsp. *arvense*	Q4	India
18	PI 166082	*P.s.* subsp. *humile*	Q6	India
19	PI 195405	*P.s.* subsp. *arvense*	Adm.	Guatemala
20	PI 203065	*P.s.* subsp. *jomardii*	Adm.	Finland
21	PI 204305	*P.s.* subsp. *sativum*	Adm.	Australia
22	PI 204667	*P.s.* subsp. *sativum*	Q3	Netherland
23	PI 220175	*P.s.* subsp. *humile*	Q6	Afghanistan
24	PI 220673	*P.s.* subsp. *arvense*	Adm.	Afghanistan
25	PI 222069	*P.s.* subsp. *humile*	Q6	Afghanistan
26	PI 234262	*P.s.* subsp. *sativum*	Adm.	USA
27	PI 254625	*P.s.* subsp. *jomardii*	Adm.	Finland
28	PI 254626	*P.s.* subsp. *jomardii*	Adm.	Australia
29	PI 261678	*P.s.* subsp. *jomardii*	Adm.	Netherland
30	PI 262189	*P.s.* subsp. *arvense*	Adm.	Costa Rica
31	PI 266069	*P.s.* subsp. *sativum*	Adm.	Sweden
32	PI 269760	*P. sativum* "Indian ecotype"	Adm.	UK
33	PI 269763	*P.s.* subsp. *jomardii*	Q2	UK
34	PI 269786	*P.s.* subsp. *sativum*	Adm.	UK
35	PI 272143	*P.s.* subsp. *jomardii*	Adm.	Germany
36	PI 272151	*P.s.* subsp. *arvense*	Adm.	Germany
37	PI 272153	*P.s.* subsp. *jomardii*	Q2	Germany
38	PI 272156	*P.s.* subsp. *jomardii*	Adm.	Peru
39	PI 280621	*P.s.* subsp. *sativum*	Q3	USSR
40	PI 280623	*P.s.* subsp. *jomardii*	Adm.	Poland
41	PI 306592	*P.s.* subsp. *jomardii*	Q2	Hungary

42	PI 312136	*P.s.* subsp. *arvense*	Adm.	Guatemala
43	PI 319373	*P.s.* subsp. *jomardii*	Adm.	Mexico
44	PI 326194	*P.s.* subsp. *jomardii*	Adm.	Mexico
45	PI 343326	*P. sativum* "Indian ecotype"	Q5	USA
46	PI 343329	*P.s.* subsp. *sativum*	Adm.	USA
47	PI 343935	*P.s.* subsp. *arvense*	Adm.	Ethiopia
48	PI 343962	*P.s.* subsp. *arvense*	Adm.	Turkey
49	PI 343965	*P.s.* subsp. *arvense*	Adm.	Turkey
50	PI 343965	*P.s.* subsp. *jomardii*	Adm.	Turkey
51	PI 343969	*P.s.* subsp. *sativum*	Q3	Turkey
52	PI 343981	*P.s.* subsp. *sativum*	Q3	Turkey
53	PI 343984	*P.s.* subsp. *arvense*	Adm.	Turkey
54	PI 343993	*P.s.* subsp. *jomardii*	Adm.	Turkey
55	PI 347282	*P. sativum* "Indian ecotype"	Q5	India
56	PI 347316	*P.s.* subsp. *arvense*	Q4	India
57	PI 347317	*P. sativum* "Indian ecotype"	Q5	India
58	PI 347319	*P.s.* subsp. *arvense*	Q4	India
59	PI 347321	*P. sativum* "Indian ecotype"	Q5	India
60	PI 347323	*P. sativum* "Indian ecotype"	Q5	India
61	PI 347326	*P. sativum* "Indian ecotype"	Q5	India
62	PI 347328	*P.s.* subsp. *arvense*	Adm.	India
63	PI 347330	*P. sativum* "Indian ecotype"	Q5	India
64	PI 347332	*P. sativum* "Indian ecotype"	Q5	India
65	PI 347333	*P. sativum* "Indian ecotype"	Q5	India
66	PI 347334	*P. sativum* "Indian ecotype"	Adm.	India
67	PI 347335	*P. sativum* "Indian ecotype"	Q5	India
68	PI 347336	*P. sativum* "Indian ecotype"	Q5	India
69	PI 347338	*P.s.* subsp. *arvense*	Adm.	India
70	PI 347342	*P.s.* subsp. *arvense*	Q4	India
71	PI 347343	*P.s.* subsp. *arvense*	Adm.	India
72	PI 343332	*P. sativum* "Indian ecotype"	Q5	India
73	PI 343333	*P. sativum* "Indian ecotype"	Q5	India
74	PI 347356	*P. sativum* "Indian ecotype"	Q5	India
75	PI 347357	*P. sativum* "Indian ecotype"	Q5	India
76	PI 347359	*P. sativum* "Indian ecotype"	Q5	India
77	PI 347366	*P.s.* subsp. *arvense*	Q4	India
78	PI 347367	*P. sativum* "Indian ecotype"	Q5	India
79	PI 347370	*P. sativum* "Indian ecotype"	Q5	India
80	PI 347372	*P.s.* subsp. *arvense*	Q4	India
81	PI 347373	*P.s.* subsp. *arvense*	Q4	India
82	PI 347374	*P.s.* subsp. *arvense*	Q4	India
83	PI 347375	*P.s.* subsp. *arvense*	Q4	India
84	PI 347383	*P.s.* subsp. *arvense*	Q4	India
85	PI 347385	*P. sativum* "Indian ecotype"	Q5	India
86	PI 347388	*P.s.* subsp. *arvense*	Q4	India
87	PI 347389	*P.s.* subsp. *arvense*	Adm.	India
88	PI 347401	*P. sativum* "Indian ecotype"	Adm.	India
89	PI 347471	*P.s.* subsp. *sativum*	Adm.	India
90	PI 358642	*P.s.* subsp. *sativum*	Q3	Ethiopia
91	PI 379612	*P.s.* subsp. *jomardii*	Adm.	Sweden
92	PI 385981	*P.s.* subsp. *sativum*	Q3	Kenya
93	PI 399129	*P.s.* subsp. *sativum*	Q3	Germany
94	PI 494079	*P.s.* subsp. *sativum*	Adm.	Chile
95	PI 560065	*P. fulvum*	Q1	Israel
96	PI 560067	*P. fulvum*	Q1	Israel
97	PI 595933	*P. fulvum*	Q1	Australia
98	PI 595945	*P. fulvum*	Q1	Jordan
99	PI 595947	*P. fulvum*	Q1	Australia
100	JI 85	*P.s.* subsp. *humile*	Q6	Afghanistan
101	JI 156	*P.s.* subsp. *jomardii*	Q2	Sudan
102	JI 156	*P.s.* subsp. *jomardii*	Q2	Sudan
103	JI 262	*P.s.* subsp. *elatius*	Q1	Turkey
104	JI 263	*P.s.* subsp. *jomardii*	Adm.	Unknown
105	JI 228	*P.s.* subsp. *arvense*	Adm.	Bolivia

106	JI 209	*P.s.* subsp. *jomardii*	Adm.	India
107	JI 209	*P.s.* subsp. *jomardii*	Adm.	India
108	JI 207	*P.s.* subsp. *jomardii*	Adm.	Russia
109	JI 224	*P. fulvum*	Q1	Israel
110	JI 196	*P.s.* subsp. *humile*	Adm.	Georgia
111	JI 190	*P.s.* subsp. *jomardii*	Q2	Sudan
112	JI 189	*P.s.* subsp. *jomardii*	Q2	Sudan
113	JI 185	*P.s.* subsp. *jomardii*	Q2	Sudan
114	JI 267	*P.s.* subsp. *jomardii*	Q2	Greece
115	JI 268	*P.s.* subsp. *jomardii*	Adm.	Crete
116	JI 275	*P.s.* subsp. *arvense*	Adm.	Crete
117	JI 280	*P.s.* subsp. *jomardii*	Adm.	Albania
118	JI 288	*P.s.* subsp. *arvense*	Adm.	Greece
119	JI 502	*P.s.* subsp. *sativum*	Q3	Unknown
120	JI 701	*P.s.* subsp. *arvense*	Adm.	Italy
121	JI 1030	*P.s.* subsp. *arvense*	Adm.	Iran
122	JI 1057	*P.s.* subsp. *sativum*	Q3	Colombia
123	JI 1089	*P.s.* subsp. *jomardii*	Adm.	Turkey
124	JI 1107	*P.s.* subsp. *humile*	Q6	Nepal
125	JI 1213	*P.s.* subsp. *sativum*	Q3	Unknown
126	JI 1345	*P.s.* subsp. *jomardii*	Adm.	Mongolia
127	JI 1346	*P.s.* subsp. *humile*	Q6	Mongolia
128	JI 2263	*P.s.* subsp. *jomardii*	Adm.	Unknown
129	JI 2265	*P.s.* subsp. *jomardii*	Adm.	Albania
130	JI 2356	*P.s.* subsp. *arvense*	Adm.	Nepal
131	JI 2385	*P. abyssinicum*	Q1	Yemen
132	JI 2387	*P.s.* subsp. *arvense*	Adm.	Ethiopia
133	JI 2545	*P.s.* subsp. *humile*	Q6	Pakistan
134	BGE001004	*P.s.* subsp. *jomardii*	Adm.	Spain
135	BGE001034	*P.s.* subsp. *sativum*	Q3	Spain
136	BGE001121	*P.s.* subsp. *sativum*	Q3	Spain
137	BGE001121	*P.s.* subsp. *sativum*	Q3	Spain
138	BGE001662	*P.s.* subsp. *jomardii*	Adm.	Spain
139	BGE002168	*P.s.* subsp. *jomardii*	Adm.	Spain
140	BGE002168	*P.s.* subsp. *jomardii*	Adm.	Spain
141	BGE003315	*P.s.* subsp. *arvense*	Adm.	Spain
142	BGE004710	*P. sativum* "Indian ecotype"	Adm.	Portugal
143	BGE004713	*P.s.* subsp. *jomardii*	Adm.	Portugal
144	BGE004958	*P.s.* subsp. *jomardii*	Adm.	Portugal
145	BGE006125	*P.s.* subsp. *arvense*	Adm.	Portugal
146	BGE006126	*P.s.* subsp. *sativum*	Adm.	Portugal
147	BGE019594	*P.s.* subsp. *arvense*	Adm.	Spain
148	BGE022159	*P.s.* subsp. *jomardii*	Adm.	Spain
149	BGE020326	*P.s.* subsp. *arvense*	Adm.	Spain
150	BGE023256	*P.s.* subsp. *jomardii*	Adm.	Spain
151	BGE025263	*P.s.* subsp. *sativum*	Adm.	Spain
152	BGE025267	*P.s.* subsp. *jomardii*	Adm.	Spain
153	BGE025270	*P.s.* subsp. *jomardii*	Adm.	Spain
154	BGE026428	*P.s.* subsp. *sativum*	Q3	Spain
155	BGE026429	*P.s.* subsp. *arvense*	Adm.	Spain
156	CGN16690	*P.s.* subsp. *jomardii*	Adm.	Italy
157	CGN03277	*P.s.* subsp. *humile*	Q6	Pakistan
158	CGN13253	*P.s.* subsp. *arvense*	Adm.	Ethiopia
159	CGN16640	*P.s.* subsp. *jomardii*	Adm.	Sudan
160	CGN16562	*P.s.* subsp. *humile*	Adm.	Mongolia
161	CGN16571	*P.s.* subsp. *jomardii*	Q2	Egypt
162	CGN16581	*P.s.* subsp. *jomardii*	Adm.	Afghanistan
163	CGN16639	*P.s.* subsp. *arvense*	Adm.	Ethiopia
164	CGN16679	*P.s.* subsp. *jomardii*	Adm.	Russia
165	CGN16582	*P.s.* subsp. *humile*	Q6	Nepal
166	CGN16684	*P.s.* subsp. *jomardii*	Adm.	Greece
167	CGN16646	*P.s.* subsp. *humile*	Adm.	Mongolia
168	CGN16636	*P. abyssinicum*	Q1	Ethiopia
169	CGN03328	*P.s.* subsp. *humile*	Q6	Pakistan

170	CGN03170	*P.s.* subsp. *arvense*	Q4	Irak
171	CGN03190	*P.s.* subsp. *jomardii*	Adm.	Turkey
172	CGN03245	*P.s.* subsp. *arvense*	Adm.	Ethiopia
173	CGN03165	*P.s.* subsp. *jomardii*	Adm.	Turkey
174	CGN03289	*P.s.* subsp. *humile*	Q6	Pakistan
175	CGN03171	*P.s.* subsp. *jomardii*	Adm.	Turkey
176	CGN03290	*P.s.* subsp. *humile*	Q6	Pakistan
177	CGN03305	*P.s.* subsp. *humile*	Q6	Pakistan
178	CGN02921	*P.s.* subsp. *sativum*	Q3	Italy
179	CGN03003	*P.s.* subsp. *sativum*	Q3	France
180	CGN03273	*P.s.* subsp. *arvense*	Adm.	Peru
181	CGN03229	*P.s.* subsp. *arvense*	Q4	Ethiopia
182	PI 413686	*P.s.* subsp. *sativum*	Adm.	Hungary
183	PI 477371	*P.s.* subsp. *jomardii*	Adm.	Denmark
184	PI 307666	*P.s.* subsp. *jomardii*	Adm.	Costa Rica
185	PI 307666	*P.s.* subsp. *jomardii*	Adm.	Costa Rica
186	PI 324693	*P.s.* subsp. *sativum*	Q3	Hungary
187	PI 324705	*P.s.* subsp. *jomardii*	Adm.	France
188	PI 355905	*P.s.* subsp. *sativum*	Q3	Japan
189	PI 241593	*P.s.* subsp. *arvense*	Adm.	China
190	PI 273207	*P.s.* subsp. *elatius*	Q1	Bulgaria
191	PI 266070	*P.s.* subsp. *jomardii*	Adm.	Sweden
192	PI 198074	*P.s.* subsp. *jomardii*	Adm.	Sweden
193	PI 357292	*P.s.* subsp. *sativum*	Q3	Serbia
194	PI 357293	*P.s.* subsp. *sativum*	Q3	Serbia
195	PI 249645	*P.s.* subsp. *arvense*	Adm.	India
196	PI 357048	*P.s.* subsp. *humile*	Q6	India
197	PI 357289	*P.s.* subsp. *sativum*	Q3	Macedonia
198	PI 253968	*P.s.* subsp. *humile*	Q6	Afghanistan
199	PI 103058	*P.s.* subsp. *sativum*	Adm.	China
200	PI 180329	*P.s.* subsp. *jomardii*	Q2	India
201	PI 184131	*P.s.* subsp. *sativum*	Q3	Serbia
202	PI 124478	*P.s.* subsp. *arvense*	Adm.	Pakistan
203	PI 124479	*P.s.* subsp. *sativum*	Adm.	Pakistan
204	PI 124479	*P.s.* subsp. *arvense*	Adm.	Pakistan
205	JI 2480	*P.s.* subsp. *jomardii*	Adm.	Peru
206	JI 1951	*P.s.* subsp. *arvense*	Adm.	China
207	JI 2302	*P.s.* subsp. *sativum*	Q3	Sweden
208	JI 1566	*P.s.* subsp. *sativum*	Q3	Unknown
209	PI 608038	*P.s.* subsp. *sativum*	Adm.	USA
210	PI 613100	*P.s.* subsp. *sativum*	Adm.	USA
211	Atc-4235-53	*P.s.* subsp. *humile*	Adm.	Australia
212	Boreen	*P.s.* subsp. *sativum*	Q3	Australia
213	Danclale	*P.s.* subsp. *jomardii*	Adm.	Australia
214	Kagpa	*P.s.* subsp. *sativum*	Adm.	Australia
215	M5	*P.s.* subsp. *sativum*	Q3	Australia
216	Pinochio	*P.s.* subsp. *sativum*	Q3	Denmark
217	B 99-114	*P.s.* subsp. *sativum*	Q3	Czech Republic
218	AGT 205,21	*P.s.* subsp. *sativum*	Q3	Czech Republic
219	Morris	*P.s.* subsp. *sativum*	Q3	Czech Republic
220	JI 1210	*P.s.* subsp. *sativum*	Q3	France
221	JI 1412	*P.s.* subsp. *sativum*	Q3	Unknown
222	JI 1559	*P.s.* subsp. *arvense*	Adm.	Mexico
223	JI 1747	*P.s.* subsp. *sativum*	Q3	Unknown
224	JI 1760	*P.s.* subsp. *sativum*	Q3	UK
225	JI 210	*P.s.* subsp. *sativum*	Adm.	Unknown
226	JI 252	*P.s.* subsp. *humile*	Q6	Ethiopia
227	JI 82	*P.s.* subsp. *humile*	Adm.	Afghanistan
228	Messire	*P.s.* subsp. *sativum*	Q3	France
229	Radley	*P.s.* subsp. *sativum*	Q3	UK
230	Ballet	*P.s.* subsp. *sativum*	Q3	UK
231	W6 17515	*P.s.* subsp. *sativum*	Q3	USA
232	W6 17516	*P.s.* subsp. *sativum*	Q3	USA
233	W6 17517	*P.s.* subsp. *sativum*	Q3	USA

234	W6 17518	*P.s.* subsp. *sativum*	Q3	USA
235	W6 17520	*P.s.* subsp. *sativum*	Q3	USA
236	KEBBY	*P.s.* subsp. *sativum*	Q3	Netherlands
237	POLAR	*P.s.* subsp. *sativum*	Q3	Poland
238	W6 17519	*P.s.* subsp. *sativum*	Adm.	Unknown
239	W6 17521	*P.s.* subsp. *sativum*	Q3	Unknown
240	BGE023667	*P.s.* subsp. *jomardii*	Adm.	Spain
241	BGE025727	*P.s.* subsp. *jomardii*	Adm.	Spain
242	PI 358608	*P.s.* subsp. *arvense*	Q4	Ethiopia
243	PI 358609	*P. abyssinicum*	Q1	Ethiopia
244	PI 173055	*P.s.* subsp. *elatius*	Adm.	Turkey
245	PI 120617	*P.s.* subsp. *elatius*	Adm.	Turkey
246	PI 273209	*P.s.* subsp. *elatius*	Adm.	Russia
247	PI 344003	*P.s.* subsp. *arvense*	Adm.	Turkey
248	PI 344005	*P.s.* subsp. *elatius*	Q1	Greece
249	PI 344006	*P.s.* subsp. *elatius*	Q1	Greece
250	PI 343976	*P.s.* subsp. *elatius*	Q1	Turkey
251	PI 505059	*P.s.* subsp. *jomardii*	Q2	Sudan
252	PI 344010	*P.s.* subsp. *elatius*	Q1	Greece
253	PI 344011	*P.s.* subsp. *elatius*	Q1	Greece
254	PI 344013	*P.s.* subsp. *elatius*	Q1	Greece
255	PI 116056	*P.s.* subsp. *arvense*	Adm.	India
256	PI 505127	*P.s.* subsp. *jomardii*	Adm.	Albania
257	PI 242027	*P.s.* subsp. *jomardii*	Q2	Denmark
258	PI 269762	*P.s.* subsp. *jomardii*	Q2	UK
259	PI 343987	*P.s.* subsp. *sativum*	Adm.	Turkey
260	PI 505080	*P.s.* subsp. *jomardii*	Adm.	Cyprus
261	PI 505111	*P.s.* subsp. *jomardii*	Adm.	Syria
262	PI 268480	*P.s.* subsp. *humile*	Q6	Afghanistan
263	JI 45	*P.s.* subsp. *humile*	Adm.	Georgia
264	JI 198	*P.s.* subsp. *arvense*	Adm.	Israel
265	JI 199	*P.s.* subsp. *arvense*	Adm.	Israel
266	JI 225	*P. abyssinicum*	Q1	Ethiopia
267	JI 227	*P. abyssinicum*	Q1	Ethiopia
268	JI 241	*P.s.* subsp. *humile*	Q6	Israel
269	JI 254	*P.s.* subsp. *elatius*	Q1	Ethiopia
270	JI 804	*P.s.* subsp. *humile*	Adm.	Unknown
271	JI 1398	*P.s.* subsp. *humile*	Adm.	China
272	JI 1428	*P.s.* subsp. *humile*	Adm.	China
273	JI 1854	*P.s.* subsp. *humile*	Q6	Israel
274	JI 2116	*P.s.* subsp. *jomardii*	Adm.	Spain
275	JI 2202	*P. abyssinicum*	Q1	Yemen
276	PIS 1318/91	*P.s.* subsp. *elatius*	Adm.	Unknown
277	CGN10205	*P.s.* subsp. *elatius*	Adm.	Unknown
278	CGN10206	*P.s.* subsp. *elatius*	Adm.	Unknown
279	CGN10193	*P.s.* subsp. *jomardii*	Adm.	Unknown
280	IFPI 3365	*P.s.* subsp. *arvense*	Adm.	Turkey
281	IFPI 3370	*P.s.* subsp. *jomardii*	Adm.	Turkey
282	IFPI 387	*P.s.* subsp. *jomardii*	Adm.	USSR
283	IFPI 436	*P.s.* subsp. *jomardii*	Q2	UK
284	IFPI 2348	*P.s.* subsp. *arvense*	Q4	Ethiopia
285	IFPI 2350	*P.s.* subsp. *arvense*	Q4	Ethiopia
286	IFPI 2351	*P.s.* subsp. *arvense*	Adm.	Ethiopia
287	IFPI 2352	*P.s.* subsp. *arvense*	Q4	Ethiopia
288	IFPI 2353	*P.s.* subsp. *arvense*	Adm.	Ethiopia
289	IFPI 2354	*P.s.* subsp. *arvense*	Q4	Ethiopia
290	IFPI 2356	*P.s.* subsp. *arvense*	Q4	Ethiopia
291	IFPI 2357	*P.s.* subsp. *arvense*	Q4	Ethiopia
292	IFPI 2358	*P.s.* subsp. *arvense*	Adm.	Ethiopia
293	IFPI 2360	*P.s.* subsp. *arvense*	Q4	Ethiopia
294	IFPI 2362	*P.s.* subsp. *arvense*	Q4	Ethiopia
295	IFPI 2363	*P.s.* subsp. *arvense*	Adm.	Ethiopia
296	IFPI 2364	*P.s.* subsp. *arvense*	Q4	Ethiopia
297	IFPI 2365	*P.s.* subsp. *arvense*	Q4	Ethiopia

298	IFPI 2367	*P.s.* subsp. *arvense*	Adm.	Ethiopia
299	IFPI 2369	*P.s.* subsp. *arvense*	Adm.	Ethiopia
300	IFPI 2370	*P.s.* subsp. *arvense*	Adm.	Ethiopia
301	IFPI 2371	*P.s.* subsp. *jomardii*	Adm.	Ethiopia
302	IFPI 2372	*P.s.* subsp. *arvense*	Q4	Ethiopia
303	IFPI 2441	*P.s.* subsp. *jomardii*	Q2	Denmark
304	IFPI 2495	*P.s.* subsp. *jomardii*	Q2	UK
305	IFPI 3232	*P. fulvum*	Q1	Syria
306	IFPI 3250	*P.s.* subsp. *jomardii*	Adm.	Syria
308	IFPI 3253	*P. fulvum*	Q1	Syria
309	IFPI 3257	*P. fulvum*	Q1	Syria
310	IFPI 3260	*P. fulvum*	Q1	Syria
311	IFPI 3261	*P. fulvum*	Q1	Syria
312	IFPI 3262	*P. fulvum*	Q1	Syria
313	IFPI 3280	*P.s.* subsp. *elatius*	Q1	Syria
314	IFPI 3282	*P.s.* subsp. *elatius*	Q1	Syria
315	IFPI 3330	*P.s.* subsp. *jomardii*	Q2	Turkey
316	IFPI 3334	*P.s.* subsp. *elatius*	Q1	Turkey
319		*P.s.* subsp. *jomardii*	Adm.	Unknown
320	JI 1006	*P. fulvum*	Q1	Israel
321	Cartouche	*P.s.* subsp. *sativum*	Adm.	Unknown
325	PI 273605	*P.s.* subsp. *jomardii*	Adm.	Ecuador
326	PI 505092	*P.s.* subsp. *jomardii*	Adm.	Cyprus
327	IFPI 2355	*P.s.* subsp. *arvense*	Q4	Ethiopia
328	JI 130	*P. abyssinicum*	Q1	Ethiopia

[1] Population structure according to Rispail *et al*. 2023. The population structure was determined by STRUCTURE for K=6. Q1 to Q6 represent the six defined subpopulations within the pea collection. Accessions were assigned to specific Q if their membership percentage was greater than 60%; otherwise, they were classified as admixed (Adm.).

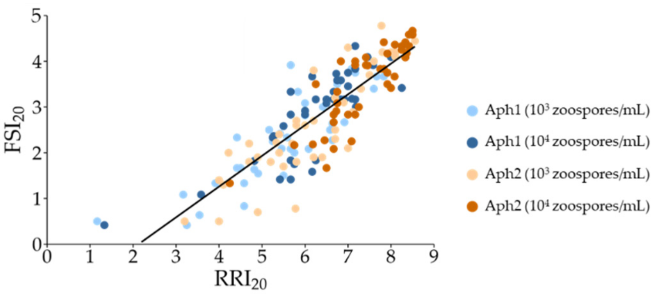

Figure S1. Relationship between Foliar Symptoms Index (FSI_{20}) (y-axis) and Root Rot Index (RRI_{20}) (x-axis) of the 40 selected accessions 20 days after the inoculation. Blue dots represent the average values of accessions inoculated with the Aph1 isolate, with light blue for the 10^3 zoospores/mL solution and dark blue for the 10^4 zoospore/mL solution. Orange dots represent the average values of accessions inoculated with the Aph2 isolate, with light orange for the 10^3 zoospores/mL solution and dark orange for the 10^4 zoospores/mL solution. The adjusted regression line is $y = 0.67 \cdot x - 1.43$ (R^2=0.75).

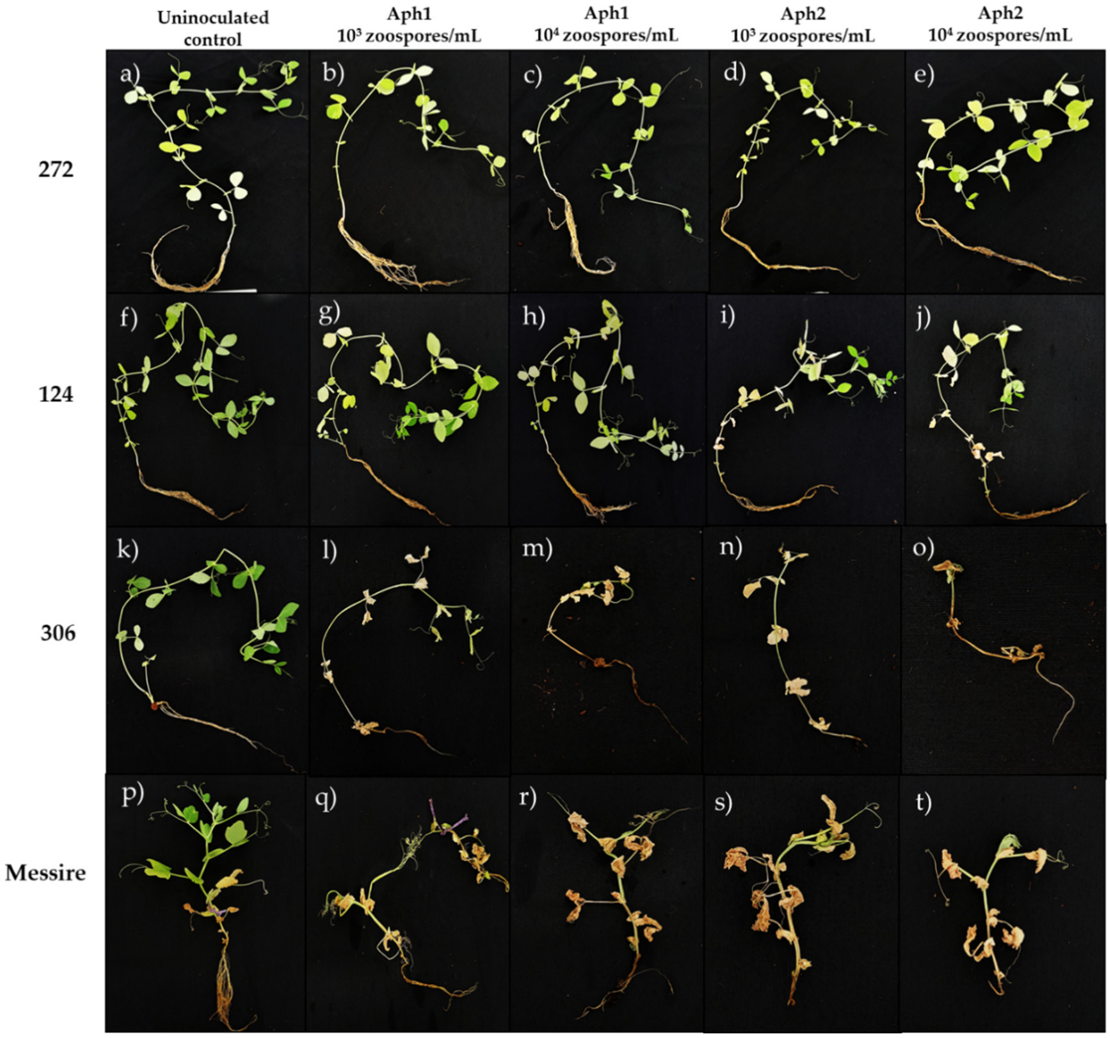

Figure S2. Phenotypic responses 20 days after the inoculation of various accessions (272, 124, 306 and the susceptible control Messire) with two isolates of *A. euteiches* (Aph1 and Aph2). **(a-t)** Individual accessions subjected to different concentrations of inoculum. Uninoculated plants are shown in the first column, followed by Aph1 inoculated at doses of 10^3 and 10^4 zoospores/mL in the second and third columns, respectively, and Aph2 inoculation at the same doses in the fourth and fifth columns, respectively. This figure visually demonstrates the range of genotype reactions, from resistance (272 and 124) to susceptibility (306) compared with the susceptible control (Messire), under experimental conditions.
